# Supplementary material for: “Mothers Should Have Freedom of Movement”—Citizens’ Attitudes Regarding Farrowing Housing Systems for Sows and Their Piglets
Source: Animals (Basel). 2021 Dec 2;11(12):3439. doi: 10.3390/ani11123439 (PMC8698166; doi:10.3390/ani11123439)
Supplement: Supplementary file 1 [file animals-11-03439-s001.zip › animals-1473074-supplementary.pdf]

Questionnaire Structure - Supplementary Material

Section 1: Research on animal husbandry

Dear participant,

This questionnaire is part of a Master's research at the Federal University of Santa Catarina on public opinion about issues in animal husbandry systems. Your participation is completely anonymous and voluntary. Only people over the age of 18 are invited to participate. You can withdraw from participation at any time by closing the questionnaire before sending it in.

( ) I agree to participate in this research

Section 2: Please fill in the following questions with your personal details

Sex:

( ) Male ( ) Female

Age:

( ) 18 to 24 years ( ) 25 to 34 years

( ) 35 to 44 years ( ) 45 to 54 years

( ) 55 to 65 years ( ) Over 65 years

Monthly income:

( ) I would rather not inform ( ) Up to 1 minimum wage

( ) 1 to 2 minimum wages ( ) 3 to 5 minimum wages

( ) 6 to 10 minimum wages ( ) Over 10 minimum wages

In which region of the country do you live?

( ) North ( ) Northeast

( ) South ( ) Southeast

( ) Midwest

Area where you live:

( ) Rural ( ) Urban

Formal education:

( ) Primary school ( ) High School

( ) Technical course ( ) Undergraduate degree

( ) Graduate degree

Do you have any kind of involvement with animal production?

( ) No

( ) Not currently, but I grew up in an environment related to animal production

( ) Yes, I am a farmer

( ) Yes, I am animal production professional

( ) Yes, I am a student of animal production

Do you have or have you ever had an animal of the following species:

	I currently have	I have had in the past	I never had
Pig	( )	( )	( )
Dog	( )	( )	( )
Cat	( )	( )	( )

Section 3: Please read the text below carefully

In production systems, the sow is housed in farrowing systems from just before farrowing until piglets are weaned. This period varies from 21 to 35 days in total. There are at least 3 types of farrowing systems. In commercial production farms, the most used system is the farrowing crate, which restricts the movement of the sow in order to avoid the death of young piglets by crushing. The loose farrowing pen and outdoor farrowing system allows the sow to move more.

In the farrowing crate system, the sow is contained inside the pen by metal bars in a space of 1.5 m^2. In this space, the sow can stand up and lie down, but cannot walk or turn around. The piglets can move freely in an area of around 4 m^2.

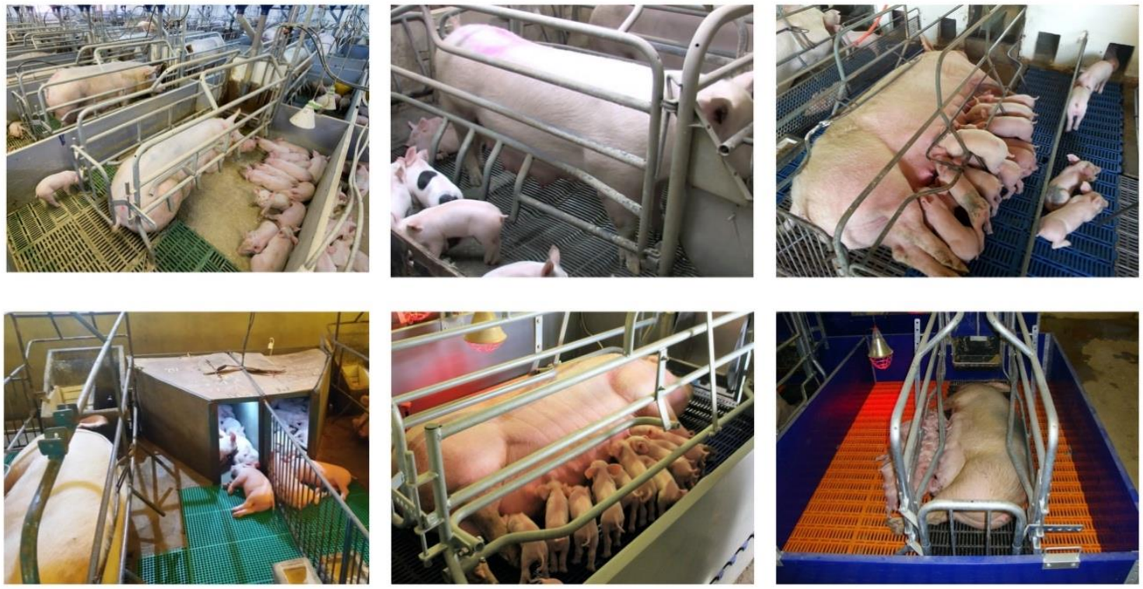

(Group of images arranged in the Farrowing Crate GIF)

In the loose farrowing pen, the animals are housed in a pen of around 6 to 9 m^2. If material like straw is added, the sow can build a nest. In this system, sow and piglets can move inside the pen.

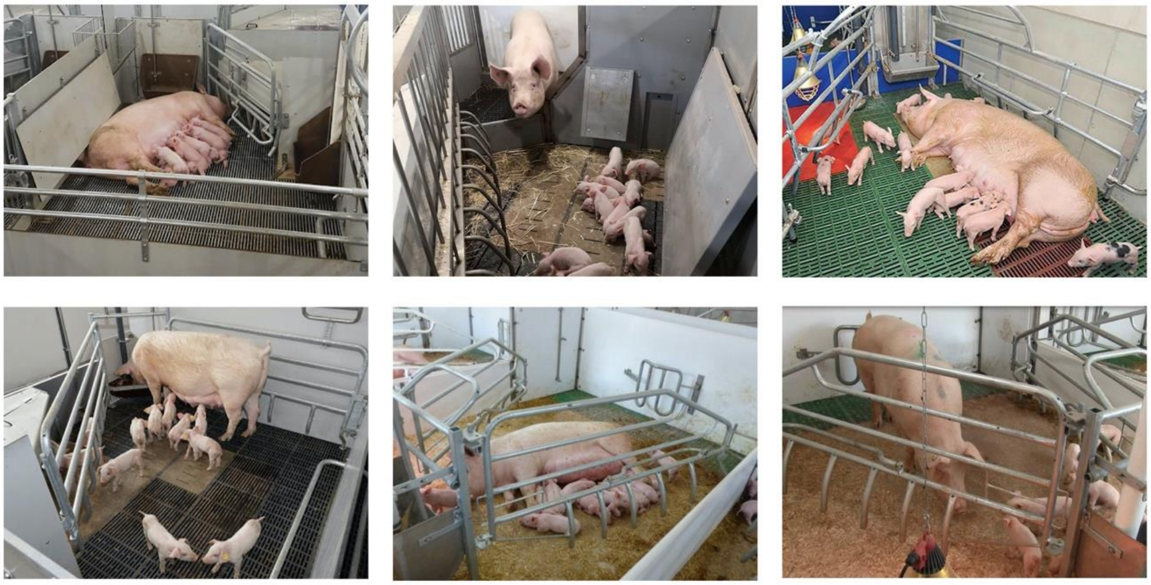

(Group of images arranged in the Loose Farrowing Pens GIF)

In the outdoor farrowing system, the animals are housed on a pasture paddock with individual huts where the sow builds her nest for farrowing. In this system sow and piglets can move around the entire grazing area.”

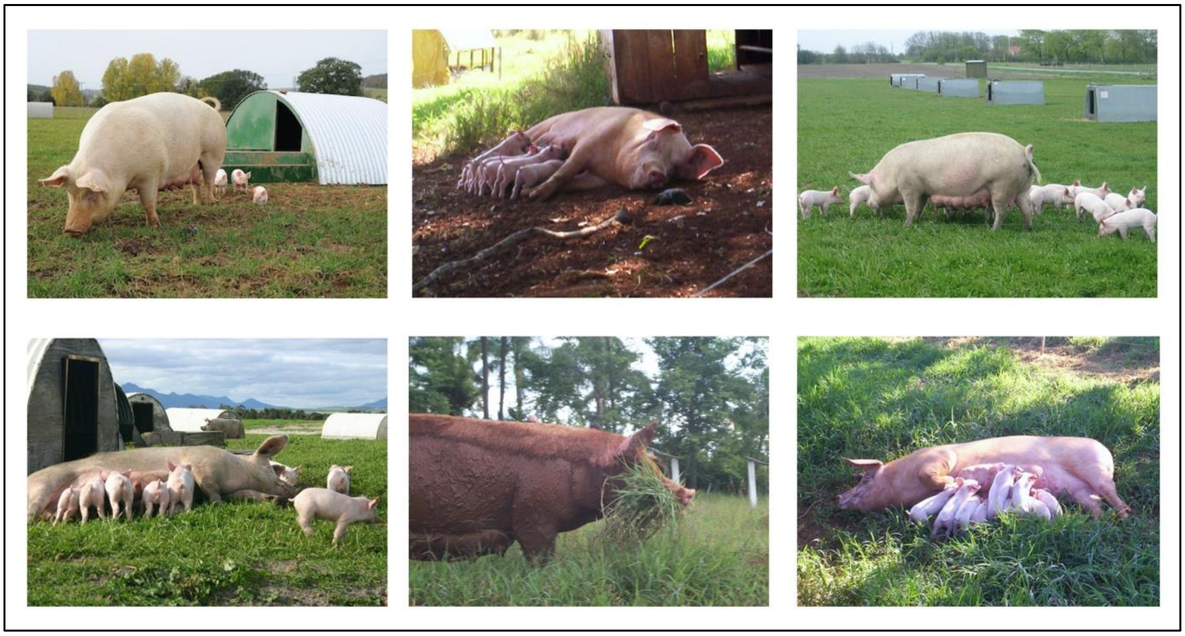

(Group of images arranged in the Outdoor Farrowing GIF)

Have you read the text above?

( ) Yes

<u>Section 4: Please answer the following questions considering the Farrowing Crate / Loose Farrowing Pens / Outdoor Farrowing</u>

(each respondent answered only about one housing system)

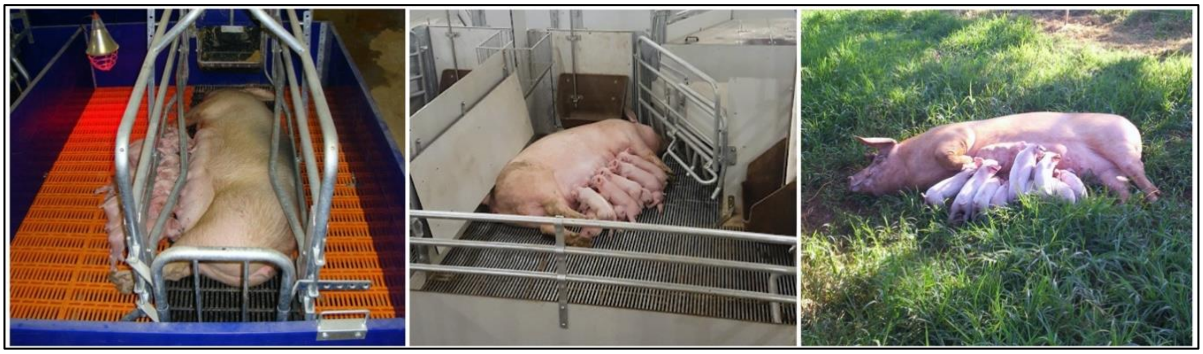

(Only the image of the system in question was displayed in this section)

Were you aware of this housing system to raise sows and piglets?

( ) Yes ( ) No

Do you consider this housing system adequate?

Totally inadequate	( )	( )	( )	( )	( )	Totally adequate

Do you approve this housing system?

Totally disapprove	( )	( )	( )	( )	( )	Totally approve

Do you consider this housing system acceptable?

Totally unacceptable	( )	( )	( )	( )	( )	Totally acceptable

Could you briefly justify your opinion about this system?

____

____

____

How do you assess the quality of life of the sows in this system?

Very bad	( )	( )	( )	( )	( )	Very good

How do you assess the quality of life of the piglets in this system?

Very bad	( )	( )	( )	( )	( )	Very good

<u>Section 5:</u>

A medium-sized company that uses farrowing crates will increase its number of sows. The company intends to adopt the loose farrowing pens for these animals. In a preliminary test of the loose farrowing pens, the company identified an increase in mortality of piglets by crushing, from 10% to 12.5%*

** Participants were randomly divided in three groups. Each group received a different type of information:*
Group 1. In a preliminary test of the loose farrowing pens, the company identified an increase in mortality of piglets by crushing, from 10% to 12.5%

Group 2. In a preliminary test of the loose farrowing pens, the company identified an increase in mortality of piglets by crushing, from 10% to 15%
Group 3. no information about piglet crushing

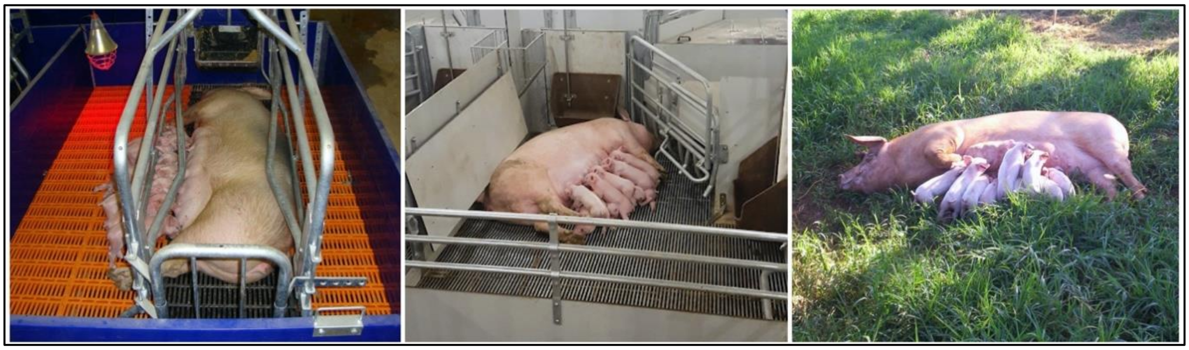

(The images of the three systems were provided in this section)

Do you believe the company should move to the loose farrowing system?

( ) Yes, I believe the company should change to the loose farrowing system

( ) No, I believe the company should keep using farrowing crates

Could you briefly justify your answer to the question above?

___

___

___

Section 6:

Based on the text previously provided in the questionnaire, indicate whether the statements below are true or false:

	True	False
The farrowing crate allows the sow to build a nest.	( )	( )
The loose farrowing pen is the most common housing system used on commercial pig farms.	( )	( )
The outdoor farrowing system allows sow and piglets to access grazing areas.	( )	( )
Choose the option 'false' to validate your answers.	( )	( )

How do you identify about rearing animals for food production?

( ) I am totally opposed to rearing animals to produce food

( ) I support rearing animals to produce food without restrictions

( ) I support rearing of animals to produce food, provided it is done in an ethical manner

Section 7: Meat consumption

Do you consume pork?

( ) Yes ( ) No

On how many days a week do you consume meat (pork, beef, chicken, fish)?

( ) None ( ) Rarely

( ) 1 to 2 days ( ) 3 to 4 days

( ) 5 to 7 days

Do you think about how animals are raised when you consume meat?

Never	( )	( )	( )	( )	( )	Always

How important is meat consumption to you?

Not important	( )	( )	( )	( )	( )	Very important

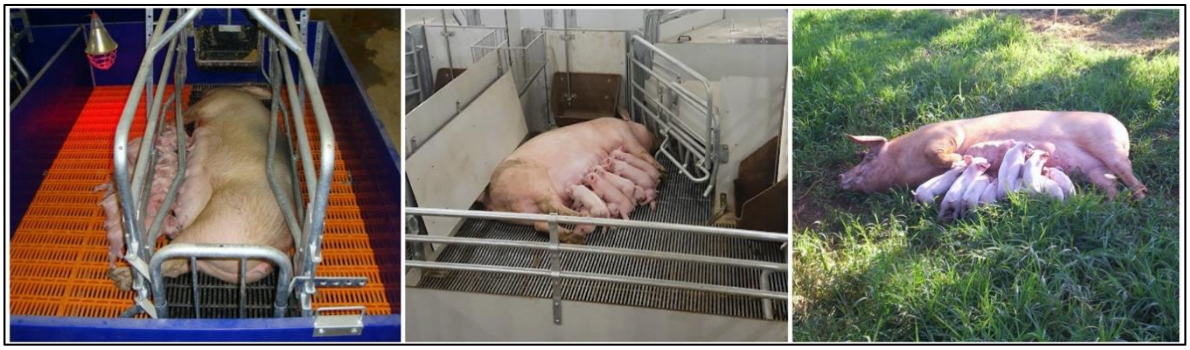

(*The images of the three systems were provided in this section*)

How much do you believe Brazilian consumers would be willing to pay more than the current price to buy pork from an animal raised in the following systems?

Loose farrowing pens: ( ) 5% more ( ) 10% more ( ) 30% more

Outdoor farrowing: ( ) 5% more ( ) 10% more ( ) 30% more

If the product had the same price, would you prefer to consume pork from an animal raised in which system?

( ) Farrowing crate

( ) Loose farrowing pen

( ) Outdoor farrowing

Could you briefly justify why you would prefer this system if the products were the same price?

___

___

___
